# Assessment of sexual and body esteem in postpartum women with or without perineal laceration: a cross-sectional study with cultural translation and validation of the Vaginal Changes Sexual and Body Esteem Scale

**DOI:** 10.61622/rbgo/2024rbgo35

**Published:** 2024-04-09

**Authors:** Renata Stefânia Olah de Souza, Adriana Gomes Luz, Ruth Zielinski, Luis Otavio Zanatta Sarian, Cassia Raquel Teatin Juliato, Lucia Alves da Silva Lara, Luiz Gustavo Oliveira Brito

**Affiliations:** 1 Universidade Estadual de Campinas Campinas SP Brazil Universidade Estadual de Campinas, Campinas, SP, Brazil.; 2 University of Michigan Michigan United States University of Michigan, Michigan, United States.; 3 Universidade de São Paulo Faculdade de Medicina de Ribeirão Preto Ribeirão Preto SP Brazil Faculdade de Medicina de Ribeirão Preto, Universidade de São Paulo, Ribeirão Preto, SP, Brazil.

**Keywords:** Postpartum, Vaginal delivery, Cesarean section, Self-concept, Sexuality

## Abstract

**Objective:**

We aimed to translate and determine cultural validity of the Vaginal Changes Sexual and Body Esteem Scale (VSBE) for Brazilian Portuguese language in postpartum women who underwent vaginal delivery with or without perineal laceration and cesarean section.

**Methods:**

A cross-sectional study conducted virtually, with online data collection through a survey with 234 postpartum women of 975 that were invited. Clinical, sociodemographic, and psychometric variables from the VSBE questionnaire were analyzed (content validity index, internal consistency, test-retest reliability, construct/structural and discriminant validity). Multivariate analysis was performed to explore associated factors with the presence of perineal laceration.

**Results:**

One-hundred fifty-eight women experienced vaginal delivery, of which 24.79% had an intact perineum, 33.33% had perineal laceration, and 9.4% underwent episiotomy; and 76 participants had cesarean sections. Women with perineal laceration were older, presented dyspareunia and previous surgeries than women without perineal laceration (p<0.05). For VSBE, a high internal consistency (Cronbach's α > 0.7) was observed, but it did not correlate with Body Attractiveness Questionnaire and Female Sexual Function Index; however, it correlated with the presence of women sutured for perineal laceration. Moreover, VSBE presented good structural validity with two loading factors after exploratory factor analysis. VSBE also demonstrated discriminant validity between the presence or absence of perineal laceration. The presence of urinary incontinence (UI) (OR=2.716[1.015-4.667];p=0.046) and a higher VSBE total score (OR=1.056[1.037-1.075];p<0.001) were the only factors associated with perineal laceration.

**Conclusion:**

Vaginal Changes Sexual and Body Esteem Scale demonstrated appropriate translation and good internal consistency, discriminant/construct validity and reliability. Vaginal Changes Sexual and Body Esteem Scale total score and presence of UI were associated with women that underwent perineal laceration.

## Introduction

The postpartum period is a phase in a woman's life that begins shortly after childbirth and extends for approximately twelve months. During this period, various physical and emotional changes occur, which, combined with the new social routine and the reality of caring for a newborn, influence postpartum women's perception of self-image, self-esteem, and sexuality.^(1,2)^ There is an expectation in the postpartum period for the body to quickly return to its pre-pregnancy appearance, with its previous shape, weight, and condition.

Excessive concern about body and genital image during sexual intercourse can compromise women's sexual health and sexuality in the postpartum period. Scarring that alters the aesthetics of the genital region, the fear of potential vaginal "looseness," and media influences all appear to be relevant factors affecting postpartum women's sexual health and sexuality.^(3-5)^ Assessing mothers in terms of perceived genital changes and postpartum genital self-image is clinically important, as the impact of these changes can adversely affect sexual health and quality of life;^(6)^ 30-70% of postpartum women reported a lower self-esteem after delivery;^(7)^ a study performed in Iran has demonstrated a reduction of feeling sexually attractive after delivery, especially women that reported vaginal laxity.^(8)^

The Vaginal Changes Sexual and Body Esteem Scale (VSBE) was developed to help healthcare professionals identify women with potential vaginal and/or anal alterations resulting from pelvic organ prolapse and was subsequently pilot tested in a population of postpartum women with perineal injuries.^(5,6)^ It is a simple, easily understood, self-administered questionnaire consisting of 10 statements about genital self-image. Translating and validating it for Brazilian Portuguese will enable better identification of low sexual esteem and altered genital self-image in this population and can enhance research in this field.

## Methods

**Figure 1:**
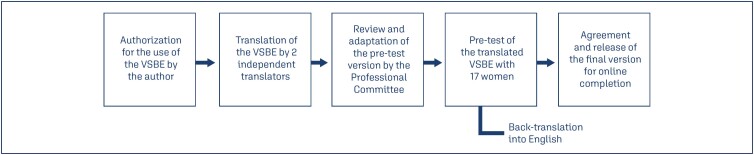
Methodological diagram of the VSBE translation and cultural adaptation process

A cross-sectional study was conducted in a virtual environment, with online data collection through a survey distributed on social media platforms (Instagram, Facebook, Twitter) and 975 women were invited. The investigators opted out to collect data in person due to the COVID-19 pandemic. The study steps are summarized in figure 1. The sample size followed the recommended range of 100 to 300 cases for validation studies; we did not perform a sample size calculation as there is considerable heterogeneity for sample size of these studies.^(9)^ Additionally, considering the suggestion of 30-60 participants for psychometric studies, 234 participants were selected as a convenience sample and subsequently categorized into four groups: cesarean section group, intact perineum vaginal delivery group, episiotomy vaginal delivery group, and perineal laceration vaginal delivery group. Although some patients knew each other, no strategy for snowball sampling was applied. A these four options were later dichotomized into two groups, as we considered episiotomy a programmed perineal laceration and women that underwent cesarean with no perineal tear.

Inclusion criteria encompassed women aged 18 years or older, primiparous individuals, within 3 to 12 months of birth at the time of survey completion and having experienced either vaginal delivery or cesarean section. Participants who did not complete all questionnaires or failed to fill out the informed consent form were excluded from the study.

Initial authorization for using the VSBE was obtained from the original author. Subsequently, the translation of the VSBE into Brazilian Portuguese was carried out by two bilingual, independent translators. One translator was Brazilian and familiar with the study's objectives, while the other translator was North American and unaware of the study's objectives. The translated versions were reviewed and adapted by a professional committee composed of three healthcare professionals, with two of them with previous experience validating more than ten health related questionnaires. A pre-test version was established, evaluated again by the expert committee, and administered to 17 women through a Google Forms questionnaire. During this stage, participants were queried about comprehension difficulties, suggestions, and critiques. However, no difficulties or suggestions were reported. A back-translation of the Brazilian Portuguese VSBE into English was performed by the same translators for comparison with the original VSBE.

The study progressed to the test and retest phase, where the initial test was administered to 234 participants along with two additional instruments for construct validity and a form for collecting clinical and sociodemographic data. The retest was conducted after 30 days, involving the same instruments and a random selection of 73 participants from the original sample of 234. Finally, the psychometric variables were used to validate the VSBE.

For the validation process, the VSBE was utilized alongside two additional instruments for comparison: the Body Attitude Questionnaire (BAQ)^(10)^ and the Female Sexual Function Index (FSFI).^(11)^

The VSBE questionnaire consists of ten questions related to self-perception and genital self-image. Each question offers responses on a Likert scale (strongly agree (1 point), agree (2 points), neutral (3 points), disagree (4 points), strongly disagree (5 points), and an option "no vaginal/rectal changes" (no value)). Final scores range from 10 to 50. Lower scores indicate more negative body and sexual self-image.^(5,6)^ In this study, only questionnaires from participants who answered all questions, excluding the option "no changes in the intimate area," were considered to calculate the minimum score.

The BAQ^(10)^ was employed in this study to estimate various aspects of postpartum women's body attitudes: physical attraction, self-deprecation, total fat, body appearance (referred to as prominence by the authors), perception of lower body fat, and strength. It comprises 44 statements, also on a Likert scale. BAQ scores range from 44 to 220 points, with higher scores indicating stronger feelings regarding the assessed aspects.

The FSFI measures female sexual response across six domains: sexual desire, sexual arousal, vaginal lubrication, orgasm, sexual satisfaction, and pain. Its 19 questions pertain to the preceding four weeks of a woman's life. The final score is the sum of points from each domain, multiplied by a domain-specific homogenizing factor. Scores range up to 36 points, with a cut-off point for sexual dysfunction considered as a score ≤ 26 points.^(11,12)^

The data were tabulated using Microsoft Excel 2013 (Richmond, VA, USA), and the analysis of sociodemographic and clinical variables was performed using the Statistical Analysis System (SAS) version 9.4 for Windows (SAS Institute Inc, 2002-2012, Cary, NC, USA). Psychometric analysis was carried out using the Intercooled Stata version 13.0 (College Station, TX, USA). Continuous variables were analyzed by the student t test for independent variables and categorical variables, by the Fisher or chi-squared test. Test-retest was calculated by paired t-test.

**Figure 2:**
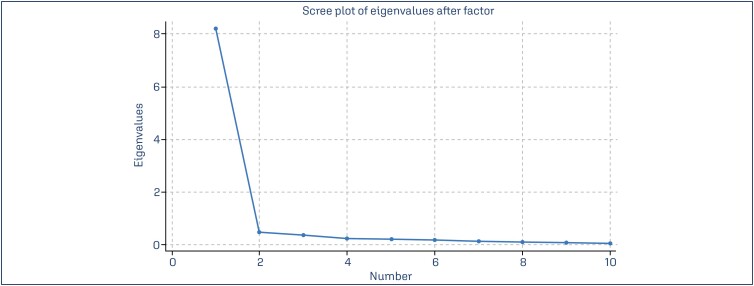
Scree plot of exploratory factor analysis for the VSBE questionnaire

The content validity index (CVI) was calculated following Lynn's guidelines,^(13)^ considering a value above 0.78 as adequate. Internal consistency of the VSBE was assessed using Cronbach's alpha coefficient, considered adequate when ≥0.70.^(14)^ Item-test correlation, item-rest correlation and covariance were also calculated for the VSBE questionnaire, and all domains and total score of the FSFI and BAQ instruments.^(15,16)^ Construct validity was assessed by correlating VSBE scores with FSFI and BAQ scores using the Spearman correlation coefficient. Moreover, exploratory factor analysis was performed considering the 10-itens of the VSBE questionnaire. The Kaiser-Meyer-Olkin (KMO) measure of sampling adequacy was performed to confirm the factorability of the data (overall=0.9447) and after finding this result, a screeplot was built (Figure 2) and revealed an inflexion point on the eigeinvalues after two loading factors. For this step, the psych and GTArotation packages of the R statistical program (R Core Team, 2021, version 4.0.4 – https://www.r-project.org) were used and the cutoff for loading factors were 0.3 for these two factors, due to their substantial contribution to data variance.

Finally, a multivariate analysis with logistic regression with all factors associated with perineal laceration was performed. A significance level of 5% was considered.

The study protocol was approved by the Institutional Review Board (CAAE: 53098521.0.0000.5404), and all participants provided informed consent before beginning the survey.

## Results

In the cross-cultural adaptation stage, the expert committee replaced the terms "vaginal/rectal area" or "vagina/rectum" with "intimate area" to cover the entire region comprising the vulva, vagina, and anus. Additionally, the insertion of the definition of the intimate area was incorporated before the scale. In the Likert scale, "Neutral" was replaced with "Indifferent" and "No vaginal/rectal changes" was replaced with "No changes in the intimate area". The translated and adapted version of the VSBE for Brazilian Portuguese is provided as supplementary material table 1S.

The Content Validity Index (CVI) of the VSBE items assessed by the expert committee is shown in supplementary material table 2S. Only item 6, related to sexual expression, did not receive the highest score in the evaluation, although it still achieved an adequate score in terms of clarity and comprehension. In total, of 975 women that were invited, 234 postpartum women participated in the survey, of whom 22 reported vaginal delivery with episiotomy, 78 had vaginal delivery with perineal laceration, 58 had vaginal delivery with intact perineum and 76 had cesarean delivery. Of the 234 puerperal women, 73 were randomly selected for retest 30 days after the initial questionnaire. These four groups were dichotomized into: Group 1, without laceration, comprising women who had normal births with an intact perineum and cesarean sections and Group 2, with laceration and/or episiotomy. The general characteristics of each group are shown in table 1. In general, women with perineal laceration did not statistically differ from women without perineal laceration regarding body mass index, race, years of education, sexual orientation, satisfaction with labor, tobacco use, presence of stable partnership, urinary incontinence, sexual activity, sexual frequency. However, women with perineal laceration were older, presented with more dyspareunia and other surgeries (cesarean, gynecological vaginal procedures) than women without perineal laceration (p<0.05).

**Table 1:** Sociodemographic and Clinical Characteristics According to the Presence of Perineal Lacerations (n=234)

Variables		Without laceration (n=100)	With laceration (n=134)	p-value
Age (years)		30.94	32.32	0.019α
Body mass index		24.46	25.53	0.113 α
Race/Ethnicity				0.763β
	White	79	108	
	Non-White	21	26	
Education				0.158 β
	>15 years	92	129	
	< 14 years	8	5	
Sexual orientation				0.953§
	Heterosexual	91	123	
	Homosexual	7	9	
	Bisexual	2	2	
Satisfaction with childbirth				0.239§
	Strongly dissatisfied	11	15	
	Dissatisfied	13	15	
	Indifferent	21	29	
	Satisfied	22	16	
	Completely satisfied	33	59	
Smoker				0.246 β
	Yes	1	0	
	No	99	134	
Stable partnership				0.250 β
	Yes	95	131	
	No	5	3	
Urinary incontinence				0.066β
	Yes	28	24	
	No	72	110	
Sexual activity				0.089β
	Yes	90	110	
	No	10	24	
Dyspareunia				<0.001β
	Yes	14	70	
	No	75	75	
Sexual frequency				0.380§
	None	17	17	
	Daily or Weekly	41	63	
	Fortnightly (Every Two Weeks)	16	28	
	Monthly	9	20	
	Other/Rarely	6	17	
Prior surgery				0.001§
	None	43	97	
	Cesarean	39	29	
	Cesarean + Other	3	5	
	Other	4	14	

α Student t-test

β Fisher test

§ Chi-square test

Table 2 shows the mean VSBE, FSFI and BAQ test scores for the two groups. The only instrument that presented discriminant validity was the VSBE total score (p<0.05); there was no between groups difference in FSFI total scores or BAQ total score and domains.

**Table 2:** Measurement of VSBE, FSFI and BAQ scores and the presence of absence of perineal lacerations

Instruments	Without laceration (n=100)	With laceration (n=134)	p-value*
VSBE Total Score	29.47	12.35	<0.005
FSFI Total Score	20.45	20.84	0.7556
FSFI Desire	2.66	2.83	0.2438
FSFI Excitation	3.35	3.25	0.6952
FSFI Lubrication	3.39	3.48	0.7242
FSFI Orgasm	3.41	3.48	0.8041
FSFI Satisfaction	3.91	3.91	0.9662
FSFI Pain	3.71	3.85	0.6252
BAQ Total Score	123.88	126.67	0.3153
BAQ Overall Fatness	11.49	12.20	0.1196
BAQ Self-disparagement	15.65	16.11	0.5005
BAQ Strength	17.63	17.82	0.3815
BAQ Salience of	23.13	23.29	0.7986
BAQ Attractiveness	16.95	16.51	0.3229
BAQ Lower body fat	39.03	40.71	0.3334

* Student t test

The estimated internal consistency of the VSBE and the retest, as well as the other instruments was calculated by Cronbach's Alpha Coefficient with all values above ≥0.70, demonstrating excellent internal consistency (Table 3). Covariance did not vary among the questionnaires, and item-test correlation values were similar.

**Table 3:** Internal consistency (Cronbach´s alpha), item-test correlation, item-rest correlation, and covariance of the VSBE, FSFI and BAQ questionnaires

Item	n	Item-test correlation	Item-rest correlation	Covariance	Alpha
VSBE Total Score	234	0.2907	0.1718	5.7086	0.7459
VSBE 1	234	0.2637	0.2548	6.0178	0.7446
VSBE 2	234	0.2173	0.2054	6.0156	0.7445
VSBE 3	234	0.2510	0.2386	6.0060	0.7442
VSBE 4	234	0.2572	0.2446	6.0033	0.7441
VSBE 5	234	0.2684	0.2548	5.9956	0.7439
VSBE 6	234	0.3019	0.2891	5.9905	0.7437
VSBE 7	234	0.2672	0.2537	5.9969	0.7439
VSBE 8	234	0.2719	0.2584	5.9951	0.7439
VSBE 9	234	0.2672	0.2540	5.9976	0.7440
VSBE 10	234	0.2692	0.2555	5.9955	0.7439
Retest VSBE Total Score	73	0.2254	0.0569	5.8704	0.7460
FSFI Total Score	234	0.2153	0.1536	5.8875	0.7432
FSFI Desire	234	0.1512	0.1440	6.0397	0.7453
FSFI Excitement	234	0.2311	0.2194	6.0107	0.7444
FSFI Lubrication	234	0.1849	0.1716	6.0167	0.7746
FSFI Orgasm	234	0.1846	0.1711	6.0160	0.7446
FSFI Satisfaction	234	0.1609	0.1514	6.0322	0.7451
FSFI Pain	234	0.1962	0.1824	6.0122	0.7445
BAQ Total Score	234	0.2879	0.1492	5.6945	0.7492
BAQ Attractiveness	234	0.0109	-0.0115	6.0647	0.7464
BAQ Self-disparagement	234	0.1733	0.1391	5.9759	0.7440
BAQ Salience	234	0.2100	0.1775	5.9959	0.7433
BAQ Lower body fat	234	0.2039	0.1812	5.9853	0.7438
BAQ Strength	234	0.0860	0.0605	6.0337	0.7455
BAQ Overall Fatness	234	0.2299	0.1434	5.8381	0.7445

Test-retest for the VSBE and BAQ (Table 4) instruments indicated good reliability as there was no statistically significant difference between the mean values of the scores at time 1 or time 2; however, FSFI total scores were different after retest.

**Table 4:** Test-retest for the VSBE, FSFI and BAQ instruments

Instruments	Mean + Standard Deviation (SD)	Mean difference + SD	p-value*
VSBE total score	20.80+-18.29	-1.86+-17.94	0.378
Retest VSBE total score	18.94+-18.66		
FSFI total score	18.77+-10.09	2.72+-9.81	0.020
Retest FSFI total score	21.49+-9.34		
BAQ total score	123.47+-21.46	2.35+-13.15	0.130
Retest total score	125.83+-23.87		

* Paired t-test

The construct validity of the VSBE instrument with other variables and clinical variables is presented in table 5 and supplementary material table 3S.

**Table 5:** Construct validity of the VSBE total score with other instruments and clinical variables

Variables	n	p-value	R Spearman
FSFI total score	234	0.532	0.04
BAQ total score	234	0.163	-0.09
VSBE total score (retest)	73	<0.001	0.55
FSFI total score (retest)	73	0.743	-0.03
BAQ total score (retest)	73	0.188	-0.15
Was sutured for perineal laceration	115	0.045	0.18
Underwent episiotomy	158	0.205	0.10
Women satisfied with labor	234	0.467	-0.04
Sexual Frequency	234	0.041	0.13

**Figure 3:**
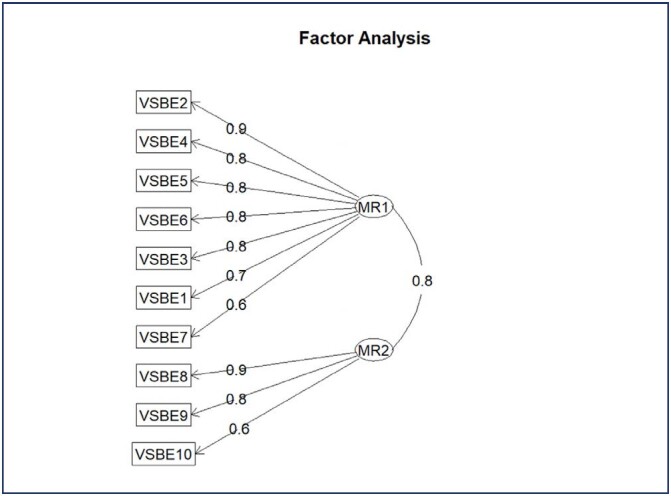
Path diagram of the revised exploratory factor analysis for the VSBE questionnaire converging the questions for two loading factors

The VSBE did not correlate with other instruments but was correlated with the presence of women that were sutured for perineal laceration and sexual frequency. Satisfaction with labor and birth was not correlated with VSBE scores. Regarding EFA, the oblique *promax* rotation was the best fit into the data (supplementary material table 3S) and has found that the first factor contributes with 42.6% of the variance and relates to questions 1 to 7 of the VSBE questionnaire; the second factor is responsible for 20.3% of the variance and relates to questions 8 to10 (Figure 3).

Finally, a multivariate analysis (Table 6) was performed to seek factors associated with perineal lacerations. Women that presented a higher VSBE score (OR=1.056[1.037-1.075]; p<0.001), and the presence of urinary incontinence (OR=2.716[1.015-4.667]; p=0.046) were associated with perineal lacerations.

**Table 6:** Multivariate analysis (logistic regression) of the associated factors with the presence of perineal lacerations

Variables	Adjusted Odds Ratio	95% CI LL-UL*	p-value**
Age < 35 years	0.502	0.222-1.137	0.099
Skin color (non-white)	1.209	0.563-2.598	0.625
Body mass index	0.951	0.878-1.030	0.220
Stable partner (yes)	0.738	0.146-3.726	0.713
Years of education	0.335	0.079-1.409	0.136
Urinary incontinence (yes)	2.176	1.015-4.667	0.046
Dyspareunia (yes)	1.832	0.952-3.523	0.070
Satisfaction with labor (yes)	1.011	0.803-1.271	0.926
VSBE total score	1.056	1.037-1.075	<0.001
BAQ total score	1.001	0.982-1.020	0.913
FSFI total score	0.987	0.952-1.024	0.497

* LL – lower limit; UL - upper limit

** Adjusted for all variables in this table

## Discussion

We demonstrated that the VSBE is a reliable questionnaire, with good internal consistency and discriminant validity between women with and without perineal laceration. However, it did not demonstrate a construct validity with a sexual function questionnaire (FSFI) and an attractiveness questionnaire (BAQ), although it correlated with the presence of laceration and most importantly, it presented two loading factors and all with values over 0.40, indicating good structural validity. Finally, multivariate analysis reported that this questionnaire and the presence of UI were associated with the presence of perineal laceration. As a research group, we perceived that the VSBE was connected to the presence of perineal laceration. This result is different from a previous study using VSBE for postpartum women, when anal sphincter tear was not associated with sexual/body esteem.^(5)^

Conversely, the same study found an association with episiotomy and lower VSBE scores, and we found higher VSBE scores, but this was not statistically significant, and this result is somewhat not expected.

In the original study involving postpartum women,^(5)^ the sample comprised 69 participants at risk of obstetric anal sphincter injuries (OASIS) (episiotomy, significant perineal lacerations, instrumental delivery). They underwent physical examination and magnetic resonance imaging to study pelvic structures. Due to the recommended social distancing measures during the Covid-19 pandemic, replicating these original study characteristics was unfeasible.

Nevertheless, our study included a sample of 234 women, with 134 adequately scoring on the VSBE, enabling a more comprehensive analysis of the Brazilian version. This study is the first translation and validation of the VSBE questionnaire into a language other than English, precluding direct comparison of our findings with other studies validating this instrument.

It is interesting to realize that self-esteem is a variable that seems to be independent of attractiveness or sexual function, in the same manner body image is. A study that validated an instrument for body image and sexual function (Body Image in the Pelvic Organ Prolapse) within women with genital prolapse found that these patients may not relate sexual function or attractiveness to POP extension. However, an impaired body image is associated with worse perception of attractiveness and increased risk for sexual dysfunction.^(17)^ A case-control study has found that women with Mayer-Rokitansky-Kuster-Hauser (MRKH) syndrome with a surgically or non-surgically created neovagina presented more dyspareunia but did not differ in overall sexual functioning from control women. Sexual esteem was significantly associated with the presence of clinically relevant sexual distress.^(18)^ A cross-sectional study with pregnant women has found that within women whose husbands displayed negative attitudes towards their weight gain during pregnancy, there was a negative relationship between depression and self-esteem scores (p<0.05), a positive correlation between self-esteem and body image scores and a negative correlation between their body image and depression scores.^(19)^

In multivariate analysis, urinary incontinence was risk factor for women with perineal laceration. We did not make a subgroup analysis to separate non-severe from severe perineal tears, as it is known from the literature that third/fourth degree injuries are associated with UI. However, there is some studies that have shown this association for second-degree tears, as a prospective cohort with 776 primiparas recently published.^(20)^

Limitations of this study were: a shorter interval for test-retest analysis would be probably more favorable than a four-week interval, selection bias as this was an online recruitment, response bias as this was an on-line study and data from these patients could not be objectively confirmed, such as presence of episiotomy. Strengths of this study is the presence of two validated instruments to compare with the VSBE, the exploratory factor analysis, a high content validity index between professionals to construct the translated questionnaire, the possibility of analyzing these patients according to the delivery route (vaginal with perineal laceration, vaginal with episiotomy, vaginal with perineal integrity and cesarean section), and as we performed an online recruitment, we presented responses from all regions of a continental country, and this provides to this study some external validity. Moreover, this is the first study performing exploratory factor analysis for this tool and two main loading factors were identified; this might suggest two domains for the instrument that could be potentially explored with future studies.

## Conclusion

The translation and cultural validation of the VSBE for Brazilian Portuguese language were carried out and the questionnaire presents good internal consistency, test-retest, discriminant, and construct validity. VSBE is strongly correlated with the presence of perineal laceration, and this tool might be useful for women that has undergone such event during labor.

## Appendix

**Supplementary material table 1S:** Vaginal Changes Sexual and Body Esteem Scale - Brazilian Version

	Concordo plenamente	Concordo	Indiferente	Discordo	Discordo completamente	Não tive alterações na área íntima
1. Sinto que as alterações em minha área íntima podem interferir no meu prazer sexual	1	2	3	4	5	N/A
2. Se eu estivesse procurando um(a) parceiro(a) sexual, acho que poderia ser mais difícil encontra-lo(a) devido às mudanças em minha área íntima	1	2	3	4	5	N/A
3. Gostaria de esconder as alterações da minha área íntima o máximo possível	1	2	3	4	5	N/A
4. Me sinto sexualmente frustrada devido às mudanças em minha área íntima	1	2	3	4	5	N/A
5. Sinto que as mudanças em minha área íntima provavelmente vão me impedir de satisfazer um(a) parceiro(a) sexual	1	2	3	4	5	N/A
6. Sinto que minha expressão sexual poderia ser limitada pelas mudanças em minha área íntima	1	2	3	4	5	N/A
7. Sinto que um(a) parceiro(a) sexual poderia não estar tão sexualmente interessado(a) em mim por causa das mudanças em minha área íntima	1	2	3	4	5	N/A
8. Eu invejo pessoas com área íntima inalterada pós parto	1	2	3	4	5	N/A
9. Se eu pudesse, eu mudaria minha área íntima por uma área íntima sem alterações	1	2	3	4	5	N/A
10. Acredito que posso sofrer rejeição de um(a) parceiro(a) sexual por causa das alterações em minha área íntima	1	2	3	4	5	N/A

TOTAL: ______________

N/A: Questão não aplicada a mim

* Área íntima: região composta por vulva (região com pelos, lábios, clitóris e entrada da vagina), canal vaginal e ânus

**Supplementary material table 2S:** Content Validity Index of VSBE Items Evaluated by the Professional Committee

Item	Professional 1	Professional 2	Professional 3	CVI
1	4	4	4	1
2	4	4	4	1
3	4	4	4	1
4	4	4	4	1
5	4	4	4	1
6	3	4	3	0.83
7	4	4	4	1
8	4	4	4	1
9	4	4	4	1
10	4	4	4	1
Mean CVI				0.98

**Supplementary material table 3S:** Factor Loadings (Pattern Matrix) And Unique Variances

VSBE questionnaire	Factor 1	Factor 2	Uniqueness
1. I feel that changes in my intimate area can interfere with my sexual pleasure	0.6341	0.1643	0.4106
2. If I were looking for a sexual partner it may be harder to find one because I have these kinds of changes to my intimate area	0.7822	0.1166	0.2341
3. I would like to hide changes in my intimate área as much as possible	0.6896	0.2395	0.2128
4. I feel sexually frustrated due to changes in my intimate area	0.7419	0.2511	0.0999
5. I feel that changes in my intimate area are likely to prevent me from satisfying a sexual partner	0.7204	0.2797	0.0926
6. I feel that my sexual expression could be limited by changes in my intimate area	0.6876	0.3054	0.1107
7. I feel that a sexual partner might not be as sexually interested in me because of changes in my intimate area	0.5907	0.4027	0.1227
8. I envy people with an unaltered intimate rea after childbirth	0.1281	0.8003	0.1853
9. If I could, I would change my intimate area to an unaltered intimate area	0.1860	0.7762	0.1406
10. I believe that I may experience rejection from a sexual partner because of changes in my intimate area	0.3508	0.6499	0.1035
